# Sex Dimorphic Responses of the Hypothalamus–Pituitary–Thyroid Axis to Maternal Separation and Palatable Diet

**DOI:** 10.3389/fendo.2019.00445

**Published:** 2019-07-11

**Authors:** Lorraine Jaimes-Hoy, Fidelia Romero, Jean-Louis Charli, Patricia Joseph-Bravo

**Affiliations:** Laboratorio de Neurobiología Molecular y Celular, Departamento de Genética del Desarrollo y Fisiología Molecular, Instituto de Biotecnología, Universidad Nacional Autónoma de México (UNAM), Cuernavaca, Mexico

**Keywords:** TRH, TRH-DE, thyroid hormones, maternal separation, stress, palatable diet, sex

## Abstract

Neonatal stress contributes to the development of obesity and has long-lasting effects on elements of the hypothalamus–pituitary–thyroid (HPT) axis. Given the importance of thyroid hormones in metabolic regulation, we studied the effects of maternal separation and a high-fat/high-carbohydrate diet (HFC), offered from puberty or adulthood, on HPT axis activity of adult male and female Wistar rats. Pups were non-handled (NH) or maternally separated (MS) 3 h/day at postnatal days (Pd) 2–21. In a first experiment, at Pd60, rats had access to chow or an HFC diet (cookies, peanuts, chow) for 1 month. Male and female NH and MS rats that consumed the HFC diet increased their caloric intake, body weight, and serum insulin levels; fat weight increased in all groups except in MS males, and serum leptin concentration increased only in females. Mediobasal hypothalamus (MBH) *Pomc* expression increased in NH-HFC females and *Npy* decreased in NH-HFC males. MS males showed insulinemia and hypercortisolemia that was attenuated by the HFC diet. The HPT axis activity response to an HFC diet was sex-specific; expression of MBH thyrotropin-releasing hormone-degrading ectoenzyme (*Trhde*) increased in NH and MS males; serum TSH concentration decreased in NH males, and T4 increased in NH females. In a second experiment, rats were fed chow or an HFC diet from Pd30 or 60 until Pd160 and exposed to 1 h restraint before sacrifice. Regardless of neonatal stress, age of diet exposition, or sex, the HFC diet increased body and fat weight and serum leptin concentration; it induced insulinemia in males, but in females only in Pd30 rats. The HFC diet's capacity to curtail the hypothalamus–pituitary–adrenal axis response to restraint was impaired in MS males. In restrained rats, expression of *Trh* in the paraventricular nucleus of the hypothalamus, *Dio2* and *Trhde* in MBH, and serum thyroid hormone concentration were altered differently depending on sex, age of diet exposition, and neonatal stress. In conclusion, metabolic alterations associated to an HFC-diet-induced obesity are affected by sex or time of exposition, while various parameters of the HPT axis activity are additionally altered by MS, pointing to the complex interplay that these developmental influences exert on HPT axis activity in adult rats.

## Introduction

Obesity is a devastating human health problem in modern societies (1). Sedentarism and stress, together with access to cheap palatable food high in fats and carbohydrates (HFC), are strong contributors whose interacting effects may synergize and provoke the metabolic syndrome (2, 3). Early-life stress due to deficient maternal care, mimicked in rodents by separation of pups from their mother, alters hypothalamus–pituitary–adrenal (HPA) axis reactivity to stress, food intake, and body weight, and impairs learning and induces anxiety and depressive behaviors (4–7). Outcomes vary among studies and may depend on time, length, and mode of pups' separation, age, or sex (4, 8–10). Daily maternal separation (MS) for 3 h during at least the first 10 postnatal days (Pd) induces long-term stress hyper-reactivity related to changes in DNA methylation of several rat or mice gene promoters involved in HPA axis activity, such as hippocampal glucocorticoid receptor (*Gr*), hypothalamic corticotrophin-releasing hormone (*Crh*) and vasopressin, and pituitary pro-opiomelanocortin (*Pomc*) (11–14). Consumption of a palatable diet rich in carbohydrates and/or fats attenuates stress response in both adult humans and rats (15–17); likewise, MS rats fed an HFC diet present a blunted HPA response to restraint stress compared to chow-fed rats (6).

The sympathoadrenal and neuroendocrine systems, including the hypothalamus–pituitary–thyroid (HPT) axis, are major regulators of energy homeostasis activating adrenergic, glucocorticoid and thyroid hormone (TH) receptors that modulate carbohydrate, lipid, and protein metabolism in multiple organs (18, 19). THs regulate basal metabolic rate accounting for 30% of energy expenditure (20) and, together with noradrenergic stimulation of brown adipose tissue, are responsible for thermogenesis (18). The important role of TH in energy homeostasis includes enhancement of lipolysis in adipose tissue followed by fatty acid uptake by active metabolic tissues, as well as increase of glucose metabolism (18, 21). The HPT axis is controlled by thyrotropin-releasing hormone (TRH) synthesized in neurons of the hypothalamic paraventricular nucleus (PVN) and released at the median eminence near portal vessels that communicate with the anterior pituitary where TRH activates TRH-R1 receptor in thyrotrophs increasing synthesis and release of thyrotropin (TSH). TSH controls the synthesis of thyroxine (T4) at the thyroid; T4 is converted to 3,3′,5-triiodo-L-thyronine (T3) by tissue deiodinases I or II (22–25). Released TRH at the median eminence may be degraded by the TRH-degrading ectoenzyme (TRH-DE), present in tanycytes, before it travels to the pituitary regulating the amount of TRH that reaches the thyrotrophs (**Figure 5A**) (26).

Chronic and some forms of acute stress inhibit various elements of the HPT axis in rats (24, 25, 27–29), and stress during critical periods of development may program its function. A history of physical/emotional abuse or neglect has been linked with reduced plasma levels of T3 in adolescents (30) and low plasma levels of TSH in adult women (31).

In a previous study, we showed that maternal separation causes long-term changes in the offspring in a sex-specific manner on some elements of the HPT axis of adult rats and blunts fasting-induced inhibition of the HPT axis activity of male rats but not females (32). Thus, we hypothesized that stress early in life may cause sex-specific responses of the HPT axis activity to an HFC diet in adult rats. Given the importance of THs in lipid and glucose metabolism as well as energy expenditure (18), the biological and physiological differences between males and females in energy metabolism (33), and especially in the neuroendocrine responses to stress (34, 35), we studied the effects of maternal separation and an HFC diet, starting at puberty or adulthood, on the HPT axis activity under basal conditions or after an acute stress insult in male and female adult rats. We also evaluated some metabolic (leptin, insulin, glucose) and neuroendocrine and endocrine (*Pomc* and *Npy* expression, HPA axis) parameters related to diet-induced obesity and stress, some of which modulate the activity of TRH neurons in the PVN.

## Materials and Methods

### Animals

**Figure 1:**
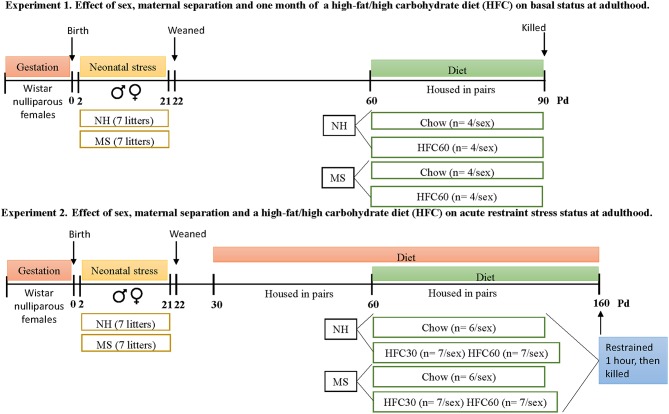
Timeline of protocols followed in experiments 1 and 2. Each experimental group from both experiments was composed by rats from a different litter, to avoid litter-mates within the same group. Abbreviations: HFC30, fed a high-fat/high-carbohydrate diet from postnatal day 30; HFC60, fed a high-fat/high-carbohydrate diet from postnatal day 60; MS, maternal separation; NH, non-handled; Pd, postnatal day.

Protocols followed the NIH Guide for the Care and Use of Laboratory Animals and the Official Mexican Norm for production, care, and use of laboratory animals NOM-062-ZOO-1999. All experiments were approved by the Bioethics Committee of the Instituto de Biotecnología, UNAM (authorization project number 311). Protocols for experiments 1 and 2 are described in Figure 1.

### Maternal Separation

Two independent experiments were performed, using a different cohort of nulliparous Wistar female rats (*n* = 14/experiment) from the Institute's outbred colony; pregnant rats were individually housed in a temperature (22 ± 1°C) and humidity (50–55%) controlled room on a 12-h dark–light cycle with chow (Teklad 2018SX, Envigo, USA) and water available *ad libitum*. As previously described (32), the day of birth was considered postnatal day 0 (Pd0); at Pd1, litters were culled to 8 pups/dam (4 males and 4 females) and were randomly assigned to the non-handled group (NH, *n* = 7 litters) for which pups were only manipulated once a week for cage cleaning or the maternal separation group (MS, *n* = 7 litters). Briefly, for the MS procedure, starting at Pd2 and until Pd21, pups were placed in a new cage with standard bedding material at 9:00 a.m. and moved to an adjacent room with controlled temperature (30–32 ± 0.5°C), 3 h/day. Pups were weaned at Pd22, and one rat from each litter was randomly selected to house four/cage according to sex and neonatal protocol (NH or MS); at Pd60, rats were housed two/cage, in separate rooms according to sex. In experiment 1, a total of 16 males and 16 females were used; in experiment 2, a total of 40 males and 40 females of a different cohort were used.

### Diet

Maternal separation affects the metabolic response to a high-fat/high-sucrose diet later in life (4) and changes some elements of the HPT axis in adult rats (32); in addition, there is a clear relationship between palatable foods high in fats and carbohydrates and stress in humans and animals that suggests a therapeutic value of “comfort foods”; in turn, stress affects feeding behavior and food choice (2, 5, 36). Thus, in the first experiment (Figure 1), we evaluated the response of the HPT axis of MS adult rats to 1 month of a palatable diet high in fat and high in carbohydrates (HFC). From Pd60 to 90, rats were randomly exposed to chow (C; NH, *n* = 4/sex; MS, *n* = 4/sex) or an HFC diet (NH, *n* = 4/sex; MS, *n* = 4/sex); all animals within each experimental group came from different litters. Basal activity of the HPT axis is inhibited in response to an acute stress (24), and the type of diet can alter the stress response of adult rats (2, 5, 36). In addition, adolescence is a vulnerable developmental period where feeding behavior is susceptible to insults (37) and availability of palatable foods increases female susceptibility to develop obesity and metabolic syndrome later in life (37, 38) and it may be exacerbated by MS in adult female rats (9). Thus, in the second experiment (Figure 1), we studied the effect of restraint stress on the activity of the HPT axis of adult obese animals with or without neonatal stress. To ensure an obese state in adult animals, chow (NH, *n* = 6/sex; MS, *n* = 6/sex) or an HFC diet (NH, *n* = 7/sex; MS, *n* = 7/sex) was offered to juvenile (Pd30) or adult (Pd60) rats for a longer period (until Pd160) compared to experiment 1. As in the first experiment, all animals within each experimental group came from different litters. NH and MS groups had *ad libitum* access to either chow (Teklad 2018SX, Envigo, USA) or an HFC diet and water. Each cage of HFC-fed rats contained three choices of food: chow [Teklad 2018SX, Envigo, USA, diet composition: 3.1 kcal/g; 21.03 g of protein, 7.01 g of fat (84% of unsaturated fatty acids, 16% of saturated fats), and 50.8 g of complex carbohydrates per 100 g], organic-animal cookies [3.9 kcal/g; 7.14 g of protein, 10.71 g of fat (66.6% of unsaturated fatty acids, 33.3% of saturated fat), and 67.85 g of carbohydrate (73.6% of complex carbohydrates, 26.3% of sugar) per 100 g; vanilla flavored, Kirkland, USA], or raw unsalted-skinned peanuts produced in Mexico [5.68 kcal/g; 25.75 g of protein, 49.4 g of fat (93.6% of unsaturated fatty acids, 6.4% of saturated fats), and 16.23 g of carbohydrate per 100 g]. Body weight was registered weekly and food intake was registered every fourth day (considering spilled food). Intake of some essential amino acids was calculated, since wheat (from cookies) is deficient in lysine, and peanuts in threonine and methionine (39, 40); digestibility and bioavailability of protein was considered (39, 41).

### Restraint Stress

To evaluate the HPT axis sensitivity of adult male and female obese rats to an acute stress with or without maternal separation, in the second experiment at Pd160, rats were restrained from 9:00 to 10:00 a.m. in a prone position, and immediately sacrificed. Since body size of rats varied, a metal wire mesh was folded according to their size, with their tail hanging out to avoid heating. A baseline blood sample was taken by tail clipping within 3 min (250–300 μl) and at 30 and 60 min following initiation of the restraint to quantify serum corticosterone levels (42). Blood glucose concentration was measured before and 60 min after introducing the rats to the restrainer using an Accutrend strip and analyzer (Roche Diagnostics, USA).

### Tissue Collection

Non-fasted animals were killed (43) at Pd90 (experiment 1) or Pd160 (experiment 2) by decapitation (42), in an independent room, by an experienced technician; trunk blood was collected to obtain the serum and was stored in aliquots at −20°C for posterior hormone determinations. Brains were immediately removed and carefully placed on crushed dry ice; once frozen, they were stored at −70°C for mRNA purification and expression analyses of genes of interest. White adipose tissue (WAT) depots (gonadal, retroperitoneal, and interscapular) were dissected and weighed fresh.

### mRNA Level Analyses

Coronal brains sections were dissected to obtain the hypothalamic PVN (Bregma −0.84 to −2.3 mm) or the mediobasal hypothalamus (MBH; Bregma −2.3 to −3.6 mm), which contains the arcuate nucleus, basal part of the third ventricle with tanycytes, and median eminence, using a 1- or a 0.5-mm-internal-diameter sample corer (Fine Science Tools, Foster City, CA), respectively. Total RNA was extracted from frozen PVN or MBH using the guanidine thiocyanate method (44); RNA concentration was quantified and its purity was verified, using a Nanodrop spectrophotometer (ThermoScientific 2000c). RNA integrity of every sample was verified by running an aliquot (0.5 μg) of intact RNA on a denaturing agarose gel; a 28S/18S rRNA ratio above 1.5 was considered adequate to use for RT-PCR. Relative mRNA levels were measured by RT-PCR; PVN *Trh* and *Crh*, and MBH *Trhde* and deiodinase type 2 (*Dio2*) mRNAs were measured as previously described (45–47). Amplification conditions for MBH *Npy* (5′-TATCCCTGCTCGTGTGTTTG-3′ and 5′-GTTCTGGGGGCATTTTCTG-3′) and *Pomc* (5′-TTGATGATGGCGTTCTTGAA-3′ and 5′-GAGATTCTGCTACAGTCGCTC-3′) cDNAs were as follows: *T*_m_ = 64°C, 26 and 27 cycles, respectively. The expression level of target genes was normalized against cyclophilin (experiment 1) or 18S ribosomal RNA (experiment 2; 5′-CGGACAGGATTGACAGATTG-3′, 5′-CAAATCGCTCCACCAACTAA-3′; *T*_m_ = 64°C and 17 cycles). Primers were synthesized in the synthesis and sequencing DNA unit of Instituto de Biotecnología, UNAM.

### Serum Hormone Analyses

Serum concentration of TSH (NIDDK reagents, Bethesda, MD; sensitivity range = 2.5–80 ng/ml) and corticosterone (reagents from Merck-Millipore, Perkin Elmer and Sigma; limits of detection, 20–2,000 ng/ml) were quantified by radioimmunoassay. Total T3 (limits of detection, 0.75–10 ng/ml) and T4 (limits of detection, 1–30 μg/dl) were quantified by ELISA [kits from Diagnóstica Internacional (Zapopan, JAL., México)] following the manufacturer's instructions, except for one variation: standard curve was prepared by adding 25 μl of rat hypothyroid serum to the calibrators provided in the kit, as recommended (48). Leptin (assay range, 0.2–12.8 ng/ml) and insulin (sensitivity range, 0.1–12.8 ng/ml) were measured using rat ELISA kits from Crystal Chem Inc. (Downers Grove, IL), ACTH using an ELISA kit from USCNK Life Science (Wuhan P.R., China; detection range, 12.35–1,000 pg/ml), and 17β-estradiol using an ELISA kit from Arbor assays (Michigan, USA; limits of detection, 3.75–120 pg/ml). Experiments 1 and 2 were analyzed independently; for each experiment, samples from male and female rats were measured in duplicate within a single assay, and inter-assay and intra-assay coefficients of variation were below 10%.

### Statistical Analyses

Results are reported as the mean ± S.E.M. Data were analyzed by a three-way ANOVA to determine effects of neonatal stress (NH vs. MS), diet (chow vs. HFC), sex (male vs. female rats), and the interaction of these variables. If a significant main effect or interaction was found, ANOVA was followed by the Holm–Sidak multiple comparisons test; the level of significance was set at *p* < 0.05. Pearson's correlation coefficient was used to examine the association between serum leptin or insulin levels and some predictive variables of obesity. Statistical analyses were performed using the GraphPad Prism8 software.

## Results

### Experiment 1. Effect of Sex, Maternal Separation, and 1 Month of an HFC Diet on Basal Status at Adulthood

#### Food Intake and Body Weight

Food intake and body weight were monitored from weaning until the end of the experiment (Pd90). There was no difference in the amount of chow consumed during Pd30–60 between MS and NH male rats (Supplementary Figure 1A), though MS males weighed slightly less than NH rats [stress effect *F*_(1, 548)_ = 4.79, *p* = 0.04; Supplementary Figure 1B]. MS did not affect chow intake and body weight of females at this age period (Supplementary Figures 1A,B). During Pd60–90, there was an effect of MS [*F*_(1, 80)_ = 6.21, *p* = 0.02], diet [*F*_(2, 80)_ = 92.83, *p* < 0.0001], sex [*F*_(1, 80)_ = 281.22, *p* < 0.0001], and an interaction of diet and sex [*F*_(2, 80)_ = 4.5, *p* = 0.04] on food intake. Chow-fed MS male rats, but not females, reduced their food intake compared to the NH group (Supplementary Figure 2A), but MS rats gained weight similarly to the NH group in both sexes; no differences were observed in intake of kcal/kg of body weight (BW) between MS-chow and NH-chow (Table 1). The HFC diet offered starting at adulthood (Pd60) for 1 month reduced chow intake in both sexes, and both NH and MS groups increased caloric intake, mainly from cookies and peanuts (Supplementary Figures 2A,B), body weight [except MS-HFC60 females; diet effect *F*_(1, 23)_ = 53.18, *p* < 0.0001; sex effect *F*_(1, 23)_ = 17.63, *p* = 0.0003; MS and diet *F*_(1, 23)_ = 5.87, *p* = 0.02; diet and sex *F*_(1, 23)_ = 6.81, *p* = 0.015; MS, diet and sex *F*_(1, 23)_ = 11.22, *p* = 0.002], and gonadal fat weight/kg [except in MS-HFC60 males; diet effect *F*_(1, 23)_ = 15.4, *p* = 0.008; Table 1]. NH and MS females had a smaller intake of total kilocalories than males [sex effect *F*_(1, 23)_ = 281.2, *p* < 0.0001], but given their lower body weight, the relative intake (kcal/kg BW) of NH-HFC60 in females was higher than in males [sex effect *F*_(1, 23)_ = 6.78, *p* = 0.015; Table 1].

**Table 1:** Effect of maternal separation and 1 month of a high-fat/high-carbohydrate diet on metabolic and HPT axis parameters (experiment 1).

	**Males**				**Females**			
	**NH**		**MS**		**NH**		**MS**	
	**C**	**HFC60**	**C**	**HFC60**	**C**	**HFC60**	**C**	**HFC60**
BWg (g)	103 ± 4	135 ± 10^*^	102 ± 4	150 ± 6^***^	29 ± 4^A^	66 ± 5^*^^A^	36 ± 5^A^	46 ± 3^A^
kcal/kg BW	202 ± 4	241 ± 6^*^	181 ± 9	225 ± 4^*^	243 ± 7	334 ± 16^***^^A^	240 ± 8	304 ± 19^*^
_G_WAT(g/kg)	45 ± 3	66 ± 6^*^	52 ± 4	61 ± 4	18 ± 4	26 ± 1.5^*^	15 ± 0.6	23 ± 0.5^*^
Leptin (ng/ml)	10.6 ± 3.5	16 ± 1.2	11.7 ± 0.4	15 ± 2	3.5 ± 0.9	9.7 ± 1.1^***^^A^	3.9 ± 0.5	8.3 ± 2^*^
Insulin (ng/ml)	1.4 ± 0.09	3.1 ± 0.6^*^	2.4 ± 0.3^η^	5 ± 1.5^*^	2 ± 0.4	3.7 ± 0.3^*^	2.2 ± 0.2	2 ± 0.09^A^
ACTH (ng/ml)	348 ± 68	346 ± 54	407 ± 8	246 ± 50	81 ± 21	35 ± 9^*^^&A^	125 ± 6^A^	144 ± 38^A^
Cort (ng/ml)	59 ± 9	56 ± 12	162 ± 26^η^	54 ± 2^*^	194 ± 25	209 ± 45^A^	174 ± 15^A^	121 ± 6
*Pomc*	100 ± 8	122 ± 14	109 ± 14	114 ± 13	100 ± 10	132 ± 6^&^*^^	111 ± 12	89 ± 13
*Npy*	100 ± 4	80 ± 2^*^	97 ± 4	86 ± 4	100 ± 1	100 ± 2	84 ± 9	96 ± 4
*Trhde*	100 ± 15	228 ± 32^*^	226 ± 36^η^	347 ± 31^*^	100 ± 13	140 ± 8^*^^A^	98 ± 10^A^	108 ± 8^A^
*Dio2*	100 ± 10	88 ± 8	91 ± 18	122 ± 6	100 ± 12	98 ± 2	109 ± 5	100 ± 6
TSH (ng/ml)	1.4 ± 0.01	0.75 ± 0.07^*^	1 ± 0.05^η^	1.2 ± 0.04	0.7 ± 0.1	0.5 ± 0.1	0.78 ± 0.1	0.56 ± 0.05
T4 (ng/dl)	2.84 ± 0.1	2.97 ± 0.3	2.48 ± 0.4	2.66 ± 0.2	1.75 ± 0.3	2.59 ± 0.3^*^	2.04 ± 0.6	2.1 ± 0.2
T3 (ng/dl)	115 ± 6	118 ± 15	105 ± 30	104 ± 8	75 ± 7	94 ± 18	93 ± 12	78 ± 1.7

*Non-handled (NH) or maternal separated (MS) male and female rats were either fed chow (C) or a high-fat/high-carbohydrate diet (HFC) from postnatal day (Pd) 60–90. Body weight gain (BWg) and intake of kcal/kg of body weight (BW) were registered during the adult period (Pd60–90), and gonadal white adipose tissue (_G_WAT) at Pd90. Metabolic and HPT axis parameters were quantified at the end of the experiment (Pd90). Expression of pro-opiomelanocortin (Pomc), neuropeptide Y (Npy), TRH degrading ectoenzyme (Trhde), and deiodinase type 2 (Dio2) were semi-quantified in the mediobasal hypothalamus (MBH) and are expressed as % of values of NH-chow rats. Data are presented as mean, S.E.M. and were analyzed by a three-way ANOVA to determine effects of neonatal stress, diet, sex, and the interaction of these variables. If a significant main effect or interaction was found, ANOVA was followed by the Holm–Sidak multiple comparisons test; the level of significance was set at p < 0.05*.

η *p < 0.05 MS-C vs. NH-C*;

* *p < 0.05*,

*** *p < 0.001 vs. chow; ^&^vs. MS-HFC60; ^A^vs. males. NH-C and NH-HFC60 n = 4/sex, MS-C and MS-HFC60 n = 4/sex. Cort, corticosterone*.

#### Neuroendocrine and Endocrine Parameters

Serum insulin levels slightly increased in MS-chow-fed males but not females [sex effect *F*_(1, 23)_ = 5.34, *p* = 0.03; Table 1], as previously described (9). After 1 month of exposure to the HFC diet, although relative epididymal WAT weight increased in NH male rats, serum leptin concentration did not change, while insulinemia was induced in NH-HFC60- and MS-HFC60-fed male rats [diet effect *F*_(1, 23)_ = 4.25, *p* = 0.05; Table 1]. Serum insulin concentration correlated positively with epididymal WAT weight of HFC-60 groups (*r* = 0.81; *p* = 0.01) but not of chow-fed males, whose epididymal WAT weight correlated instead with serum leptin concentration (*r* = 0.66; *p* = 0.03). In contrast, in NH and MS females fed an HFC diet, serum leptin concentration increased [diet effect *F*_(1, 23)_ = 22.43, *p* = 0.0002; sex effect *F*_(1, 23)_ = 5.96, *p* = 0.02; diet and sex effect *F*_(1, 23)_ = 5.51, *p* = 0.03], while insulinemia was only present in NH-HFC60 rats (Table 1).

MS induced persistent stress in chow-fed male rats, evidenced by increased serum corticosterone concentration that was lowered by 1 month of feeding an HFC diet [MS and diet effect *F*_(1, 23)_ = 6.48, *p* = 0.02; MS and sex effect *F*_(1, 23)_ = 5.82, *p* = 0.02; Table 1], but no change in serum ACTH concentration was observed, compared to NH-chow-fed rats (Table 1). In contrast, neither an HFC diet nor MS affected stress markers in adult female rats, except for a lower serum ACTH concentration in NH-HFC60 compared to chow-fed female rats [MS and sex effect *F*_(1, 23)_ = 12.11, *p* = 0.0025; MS, diet, and sex effect *F*_(1, 23)_ = 5.41, *p* = 0.03; Table 1]. Serum 17β-estradiol concentration was not affected by MS or 1 month of feeding an HFC diet in female rats (Supplementary Figure 3A).

Expression of MBH peptides was modified by sex and/or the HFC diet, but not by MS; *Pomc* mRNA levels increased in NH-HFC60 females compared to NH-chow and MS-HFC60 groups [diet effect *F*_(1, 23)_ = 5.04, *p* = 0.04; Table 1], and *Npy* mRNA levels decreased in NH-HFC60 males compared to chow-fed rats [diet effect *F*_(1, 23)_ = 5.51, *p* = 0.04; diet and sex effect *F*_(1, 23)_ = 13.5, *p* = 0.001; Table 1] as reported by Obici et al. (49) and Schwinkendorf et al. (50).

MS modified elements involved in the HPT axis activity, including an increase of *Trhde* mRNA levels in the MBH [MS effect *F*_(1, 23)_ = 6.67, *p* = 0.019; sex effect *F*_(1, 23)_ = 29.86, *p* < 0.0001; MS and sex effect *F*_(1, 23)_ = 10.28, *p* = 0.005; Table 1] and a decrease of serum TSH concentration [MS effect *F*_(1, 23)_ = 5.47, *p* = 0.04; Table 1] compared to NH males, but not in females, as published (32). Consumption of an HFC diet increased MBH-*Trhde* expression in NH and MS males [diet effect *F*_(1, 23)_ = 13.02, *p* = 0.002; diet and sex effect *F*_(1, 23)_ = 5.15, *p* = 0.03; Table 1], while only in NH-HFC60 females compared to NH-chow-fed rats (Table 1). MBH-*Dio2* expression was not modified under any condition (Table 1). The HFC diet decreased serum TSH concentration in NH males compared to chow-fed rats [diet effect *F*_(1, 23)_ = 4.31, *p* = 0.05; Table 1], whereas that of T4 was higher only in female NH-HFC60 than in NH-chow rats [diet effect *F*_(1, 23)_ = 4.28, *p* = 0.05; Table 1].

### Experiment 2. Effect of Sex, Maternal Separation, and an HFC Diet on Acute Restraint Stress Status at Adulthood

#### Food Intake and Diet Preference, Body Weight, and Composition

**Figure 2:**
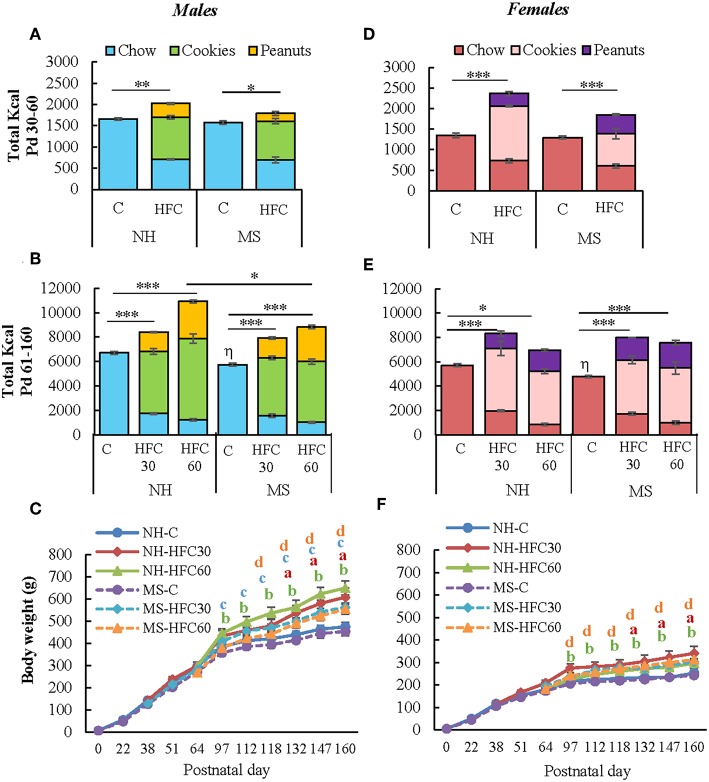
Effect of maternal separation and a high-fat/high-carbohydrate diet on food intake and body weight. Male and female pups were non-handled (NH) or separated from the dam (MS) daily from postnatal day 2–21 (Pd) for 3 h. NH and MS rats had free access to either chow (C) or a high-fat/high-carbohydrate diet (HFC: cookies, peanuts, and chow) from Pd30 (HFC30) or 60 (HFC60) until Pd160. Total kilocalorie intake during the juvenile period **(A,D)** and adulthood **(B,E)**. **(C,F)** Body weight of male and female rats from birth until Pd160. Food intake was calculated by dividing the daily kilocalorie intake per cage by the number of rats in the cage (two rats/cage). Data are expressed as mean ± S.E.M. and were analyzed by a three-way ANOVA to determine effects of neonatal stress, diet, sex, and the interaction of these variables. If a significant main effect or interaction was found, ANOVA was followed by the Holm–Sidak multiple comparisons test; the level of significance was set at *p* < 0.05. ^*^p < 0.05, ^**^p < 0.01, ^***^p < 0.001, ^η^vs. NH-C, ^a^NH-HFC30 vs. NH-C, ^b^NH-HFC60 vs. NH-C, ^c^MS-HFC30 vs. MS-C, ^d^MS-HFC60 vs. MS-C. NH-C *n* = 6/sex, NH-HFC30 and NH-HFC60 *n* = 7/sex, MS-C *n* = 6/sex, MS-HFC30, and MS-HFC60 *n* = 7/sex.

NH and MS rats were fed a chow or a high-fat/high-carbohydrate diet from Pd30 or 60 up to Pd160 and then exposed 1 h to restraint stress before sacrifice. As in experiment 1, MS males and females on a chow diet consumed similar kcal as their respective NH group during adolescence (Figures 2A,D). MS did not change food preference; however, MS-chow rats of both sexes had lower food intake than NH-chow animals during adulthood, with males consuming more kcal than females [MS effect *F*_(1, 80)_ = 10.8, *p* < 0.0014; sex effect *F*_(1, 80)_ = 65.4, *p* < 0.0001; MS and sex effect *F*_(1, 80)_ = 5.66, *p* = 0.01; Figures 2B,E]; thus, the proportion of each macronutrient contributing to total kilocalorie intake remained the same (Supplementary Table 1). Availability of palatable food diminished chow intake [diet effect *F*_(1, 80)_ = 4011.23, *p* < 0.0001; sex effect *F*_(1, 80)_ = 20.45, *p* < 0.0001; sex and diet effect *F*_(1, 80)_ = 15.45, *p* = 0.0002], with cookies being the largest contributor of kilocalorie intake in male and female rats followed by peanuts, at all ages (Figures 2A,B,D,E). NH and MS male and female rats that had access to the HFC from Pd30 (HFC30 group) consumed slightly more total kilocalories compared to chow-fed groups during the adolescence period [diet effect *F*_(1, 80)_ = 157.24, *p* < 0.0001; MS and diet effect *F*_(1, 80)_ = 11.2, *p* = 0.001; diet and sex effect *F*_(1, 80)_ = 33.97, *p* < 0.0001; Figures 2A,B]. During the adult period (Pd61–160), all NH-HFC and MS-HFC fed male and female rats consumed higher total kilocalories than chow-fed rats, but kilocalorie intake was even higher in the NH-HFC60 group compared to MS-HFC60 males [MS effect *F*_(1, 80)_ = 10.8, *p* = 0.0014; diet effect *F*_(2, 80)_ = 77.45, *p* < 0.0001; diet and sex effect *F*_(1, 80)_ = 33.97, *p* < 0.0001; Figures 2B,E].

Male and female rats fed an HFC diet, either from Pd30 or 60, ingested a similar proportion of macronutrients during Pd60–90 or Pd91–160, diminishing protein intake in favor of lipids (Supplementary Table 1). In HFC-fed animals, protein intake diminished, although to amounts and a proportion adequate for protein maintenance considering the quality of protein (51), and recommended intake of essential amino acids for growth maintenance was preserved (51) (Supplementary Table 1).

The slope of body weight gain was reduced after Pd97 in NH- and MS-chow-fed groups of both sexes while the reduction was less conspicuous in those receiving an HFC diet, especially in NH-HFC60 males compared to NH-chow and MS-HFC60 groups [diet effect *F*_(2, 80)_ = 7.18, *p* = 0.007; sex effect *F*_(1, 80)_ = 130.85, *p* < 0.0001; Figures 2C,F]. NH-chow and NH-HFC60 males consumed more kilocalories per day than females [diet effect *F*_(1, 80)_ = 21.9, *p* < 0.0001; sex effect *F*_(1, 80)_ = 65.4, *p* < 0.0001] but not relative to their body weight (Supplementary Table 1); MS-chow and MS-HFC males also consumed more kilocalories per day than females during Pd91–160 [diet and sex effect *F*_(2, 80)_ = 4.8, *p* = 0.04; Supplementary Table 1]. Food efficiency was lower in female than in male groups [diet and sex effect *F*_(2, 80)_ = 8.18, *p* = 0.003; Supplementary Table 1], as shown in adult rats (4).

Weights of gonadal fat [diet effect *F*_(2, 80)_ = 55.52, *p* < 0.0001; diet and sex *F*_(2, 80)_ = 10.98, *p* < 0.0001], retroperitoneal fat [diet effect *F*_(2, 80)_ = 66.33, *p* < 0.0001; diet and sex effect *F*_(2, 80)_ = 4.23, *p* = 0.018], and interscapular fat [diet effect *F*_(2, 80)_ = 27.25, *p* < 0.0001] increased with consumption of an HFC diet, to a similar extent whether diet started at Pd30 or 60 in NH and MS males and females compared to chow-fed rats (Table 2); MS-HFC60 female rats showed a tendency of higher interscapular WAT weight than NH-HFC60 but did not achieve statistical significance (*p* = 0.06) (Table 2).

**Table 2:** Effect of maternal separation and 3–4 months of a high-fat/high-carbohydrate diet on metabolic parameters in acutely restrained rats (experiment 2).

**Males**	**NH**			**MS**		
	**C**	**HFC30**	**HFC60**	**C**	**HFC30**	**HFC60**
Epididymal WAT (g/kg)	24.92 ± 2.6	47.4 ± 5.2^*^	43.8 ± 3.3^*^	27.54 ± 2.6	44.53 ± 4^*^	40.1 ± 3.9^*^
Retroperitoneal WAT (g/kg)	36.84 ± 4.9	70.05 ± 9.8^*^	88.02 ± 3.8^**^	37.85 ± 3.3	71.96 ± 4.8^*^	67.09 ± 5.1^*^
Interscapular WAT (g/kg)	5.43 ± 0.7	11.9 ± 3.3^*^	13.06 ± 0.4^*^	4.92 ± 0.4	9.55 ± 2.8^*^	11.1 ± 1.2^*^
Leptin (ng/ml)	6.19 ± 0.8	14.1 ± 1.5^*^	17.57 ± 1.2^*^	7.5 ± 0.7	10.7 ± 0.8^*^	14.4 ± 0.7^*^
Glucose before RES (mg/dL)	98 ± 2.2	123 ± 3.2^**^	120 ± 2.4^*^	102 ± 2.7	127 ± 3.5^**^	117 ± 2.5^*^
Glucose 60 min after RES (mg/dL)	164 ± 7.6^+^	174 ± 7.2^+^	192 ± 9.2^*^^+^	145 ± 2.6^+^	182 ± 5.7^*^^+^	185 ± 8.1^*^^+^
Insulin (ng/mL)	0.68 ± 0.04	1.07 ± 0.09^*^	1.6 ± 0.16^*^	0.63 ± 0.1	1.22 ± 0.08^*^	1.25 ± 0.1^*^
**Females**	**NH**			**MS**		
	**C**	**HFC30**	**HFC60**	**C**	**HFC30**	**HFC60**
Ovaric WAT (g/kg)	21.6 ± 2.4	71.7 ± 3.3^***^	56.73 ± 4.6^***^	27.08 ± 2.3	65.2 ± 6.2^***^	79.07 ± 9.7^***^
Retroperitoneal WAT (g/kg)	18.64 ± 1.7	60.3 ± 3.3^***^	52.24 ± 6.3^***^	24.9 ± 3	57.5 ± 4^***^	59.47 ± 4.6^***^
Interscapular WAT (g/kg)	3.91 ± 0.4	15.6 ± 3.8^**^	8.28 ± 1.5^*^	4.73 ± 0.5	15 ± 1.5^**^	15.9 ± 2.3^**^
Leptin (ng/mL)	2.47 ± 0.27	6.58 ± 0.8^*^	8.05 ± 1.13^***^	3.2 ± 0.2	9.2 ± 1.1^***^	10.26 ± 1.5^***^
Glucose before RES (mg/dL)	124 ± 5.3	119 ± 3.9	128 ± 4.6	115 ± 2.6	123 ± 4.9	120 ± 3.8
Glucose 60 min after RES (mg/dL)	162 ± 7.6^+^	161 ± 4.1^+^	169 ± 6.5^+^	141 ± 4.7^+^	165 ± 10^+^	160 ± 7.7^+^
Insulin (ng/mL)	0.1 ± 0.03	0.27 ± 0.05^*^	0.17 ± 0.01	0.13 ± 0.02	0.29 ± 0.05^*^	0.13 ± 0.01

*Non-handled (NH) or maternal separated (MS) male and female rats fed a chow (C) or a high-fat/high-carbohydrate diet (HFC) from Pd30 (HFC30) or 60 (HFC60) until Pd160 were restrained 1 h before they were killed. White adipose tissue (WAT) depots and serum concentrations of leptin and insulin were determined at the end of the experiment (Pd160). Blood glucose concentration was quantified before restraint (RES) and 60 min after RES. Data are presented as mean ± S.E.M. and were analyzed by a three-way ANOVA to determine effects of neonatal stress, diet, sex, and the interaction of these variables. If a significant main effect or interaction was found, ANOVA was followed by the Holm–Sidak multiple comparisons test, the level of significance was set at p < 0.05*.

* *p < 0.05*,

** p < 0.01, and

*** *p < 0.001 vs. respective chow group*;

+ *p < 0.01 vs. glucose before RES. NH-C n = 6/sex, NH-HFC30 and NH-HFC-60 n = 7/sex, MS-C n = 6, MS-HFC30, and MS-HFC60 n = 7/sex*.

#### Metabolic Parameters and Serum 17β-Estradiol

Serum leptin concentrations of HFC-fed NH and MS male and female rats were higher than those of NH-chow-fed rats [diet effect *F*_(2, 80)_ = 42.82, *p* < 0.0001; Table 2]. Changes in serum insulin concentration followed a similar pattern to serum leptin in males; in contrast, in females, serum insulin concentration augmented only in NH-HFC30 and MS-HFC30 compared to chow and HFC60 groups [diet effect *F*_(2, 80)_ = 10.22, *p* = 0.0001; diet and sex effect *F*_(2, 80)_ = 5.33, *p* = 0.007; Table 2]. Basal glucose levels were not changed by MS in chow-fed animals, but levels were higher in HFC-fed NH and MS rats compared to chow-fed males, regardless of the age of access to the diet and were unchanged by an HFC diet in females [diet effect *F*_(2, 80)_ = 12.03, *p* < 0.0001; sex effect *F*_(1, 80)_ = 8.77, *p* = 0.004; Table 2]. In response to restraint, blood glucose levels increased in all chow and HFC groups in both sexes and were even higher in NH-HFC60, MS-HFC30, and MS-HFC60 males in comparison to chow-fed rats, with no differences between groups in females [diet effect *F*_(2, 80)_ = 11.37, *p* < 0.0001; sex effect *F*_(1, 80)_ = 11.46, *p* = 0.001; Table 2]. The concentration of 17β-estradiol was lower after 3–4 months of feeding an HFC diet to NH and MS female rats [diet effect *F*_(2, 40)_ = 6.51, *p* = 0.003; Supplementary Figure 3B].

#### HPA Axis Parameters

**Figure 3:**
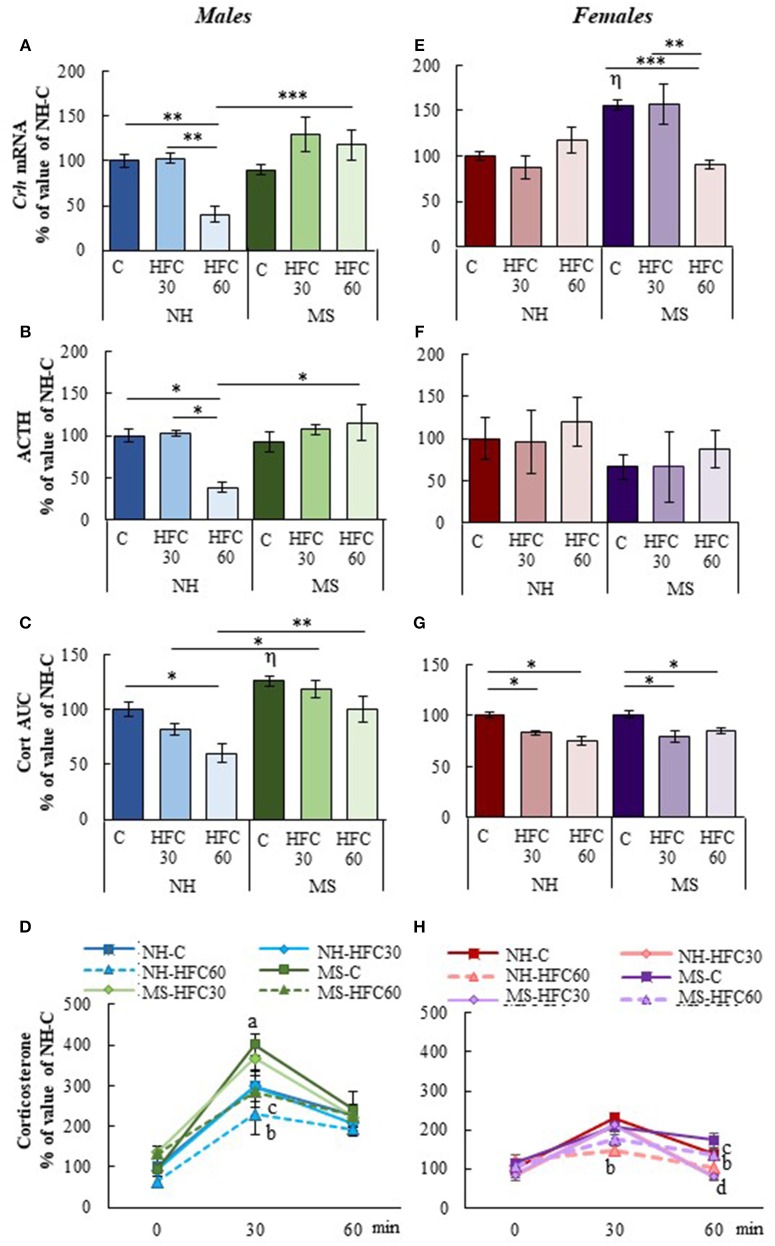
Effect of maternal separation and a high-fat/high-carbohydrate diet on HPA axis parameters in acutely restrained rats. Non-handled (NH) or maternal separated (MS) male and female rats fed a chow (C) or a high-fat/high-carbohydrate diet (HFC) from Pd30 (HFC30) or 60 (HFC60) until Pd160 were restrained 1 h before they were killed. **(A,E)** Expression levels of *Crh* in the paraventricular nucleus. **(B,F)** Serum concentration of ACTH. **(C,G)** Area under the curve (AUC) of serum corticosterone concentration response to restraint shown in **(D,H)**; serum corticosterone values are presented in % of NH-chow groups to compare the extent of the stress response between males and females, as females present higher basal levels of serum corticosterone than male rats; absolute values are shown in Supplementary Figure 4. Data are mean ± S.E.M. expressed as % of values of NH-chow rats. Data were analyzed by a three-way ANOVA to determine effects of neonatal stress, diet, sex, and the interaction of these. If a significant main effect or interaction was found, ANOVA was followed by the Holm–Sidak multiple comparisons test, the level of significance was set at *p* < 0.05. ^*^p < 0.05, ^**^p < 0.01, ^***^p < 0.001, ^η^vs. NH-C, ^a^MS vs. NH, ^b^NH-HFC60 vs. NH-C, ^c^MS-HFC60 vs. MS-C, ^d^NH- and MS-HFC30 vs. NH-C and MS-C. NH-C *n* = 6/sex, NH-HFC30 and NH-HFC60 *n* = 7/sex, MS-C *n* = 6/sex, MS-HFC30 and MS-HFC60 *n* = 7/sex.

After 1 h of restraint, serum corticosterone AUC was significantly higher in MS-chow than in NH-chow-fed male rats [MS effect *F*_(1, 80)_ = 20.5, *p* < 0.0001; Figure 3C], but serum ACTH concentration and mRNA levels of *Crh* in the PVN were unchanged (Figures 3A,B). Ingestion of an HFC diet diminished the stress response only in NH males that consumed the diet from adulthood, but not of MS males, since PVN*-Crh* expression [MS effect *F*_(1, 80)_ = 18.3, *p* < 0.0001; diet effect *F*_(2, 80)_ = 4.38, *p* = 0.016; Figure 3A], serum concentration of ACTH [MS and sex effect *F*_(1, 80)_ = 10.1, *p* < 0.002; Figure 3B], corticosterone at 30 min and corticosterone AUC were lower in NH-HFC60 than in chow-fed rats [MS effect *F*_(1, 80)_ = 20.5, *p* < 0.0001; diet effect *F*_(2, 80)_ = 17.59, *p* < 0.0001; MS and sex effect *F*_(1, 80)_ = 15.4, *p* = 0.0002; Figures 3C,D]. Thus, MS impaired the inhibitory effect of an HFC diet on the restraint-induced activation of the HPA axis in male rats. Females responded differently; MS enhanced *Crh* expression in the PVN of chow-fed rats and was reduced in MS-HFC60 but not MS-HFC30 compared to chow-fed MS rats [sex effect *F*_(1, 80)_ = 13.92, *p* = 0004; MS, diet and sex effect *F*_(2, 80)_ = 17.45, *p* < 0.0001; Figure 3E]. No changes in serum ACTH concentration were found in females (Figure 3F), whereas serum corticosterone at 30 min and AUC was similar between NH- and MS-chow-fed groups and comparable lower in all groups fed an HFC diet regardless of neonatal stress or age of diet exposition [sex effect *F*_(1, 80)_ = 6.03, *p* = 0.016; MS and sex effect *F*_(1, 80)_ = 15.4, *p* = 0.0002; Figures 3G,H].

#### Mediobasal Hypothalamic Neuropeptides and HPT Axis Parameters

**Figure 4:**
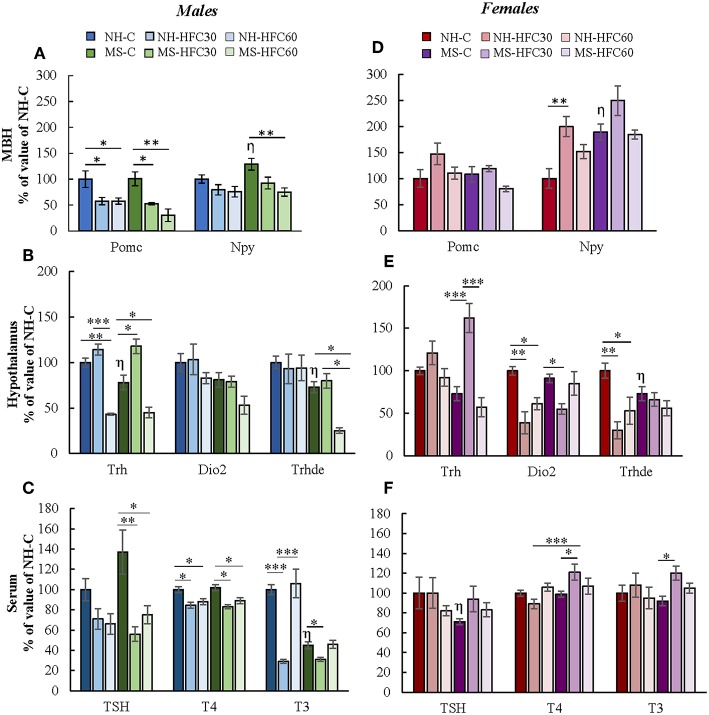
Effect of maternal separation, and a high-fat/high-carbohydrate diet on the expression of arcuate peptides and HPT axis parameters in acutely restrained rats. Non-handled (NH) or maternal separated (MS) male and female rats fed a chow (C) or a high-fat/high-carbohydrate diet (HFC) from Pd30 (HFC30) or 60 (HFC60) until Pd160 were restrained 1 h before they were killed. **(A,D)** Expression levels of pro-opiomelanocortin (*Pomc*) and neuropeptide Y (*Npy*) in the mediobasal hypothalamus (MBH). **(B,E)** Expression levels of *Trh* in the paraventricular nucleus and of *Dio2* and *Trhde* in the MBH: **(C,F)** Serum concentrations of TSH, T4, and T3. Data are expressed as % of values of NH-chow illustrated as mean ± S.E.M. Data were analyzed by a three-way ANOVA to determine effects of neonatal stress, diet, sex, and the interaction of these variables. If a significant main effect or interaction was found, ANOVA was followed by the Holm–Sidak multiple comparisons test; the level of significance was set at *p* < 0.05. ^*^p < 0.05, ^**^p < 0.01, ^***^p < 0.001,^η^vs. NH-C. NH-C *n* = 6/sex, NH-HFC30 and NH-HFC60 *n* = 7/sex, MS-C *n* = 6/sex, MS-HFC30 and MS-HFC60 *n* = 7/sex.

The PVN-*Trh* expression is regulated in part by peptides released from neurons located within the arcuate nucleus that project to TRH synthesizing neurons in the PVN of the hypothalamus, positively by POMC and negatively by NPY (22). MBH-*Pomc* expression in males was reduced by an HFC diet, with no effects of maternal separation or age of access to the HFC diet [diet effect *F*_(2, 80)_ = 4.07, *p* = 0.022; sex effect *F*_(1, 80)_ = 25.45, *p* < 0.0001; diet and sex effect *F*_(2, 80)_ = 7.2, *p* = 0.001; Figure 4A], whereas no changes were found in female rats (Figure 4D). MBH-*Npy* expression was enhanced in both MS-chow male and female rats compared to NH-chow groups (Figures 4A,D) and reduced in MS-HFC60 males compared to MS-chow-fed rats [MS effect *F*_(1, 80)_ = 22.2, *p* < 0.0001; sex effect *F*_(1, 80)_ = 74.73, *p* < 0.0001; diet and sex effect *F*_(2, 80)_ = 6.85, *p* = 0.002; Figure 4A].

In adult male rats, acute restraint is reported to reduce PVN-*Trh* expression and serum TSH concentration (46), but serum T3 concentration only after a larger restraint span (52). Various parameters of the HPT axis in response to restraint stress were altered differently according to neonatal stress, sex, and age of access to the HFC diet. In male rats, MS-chow feeding regulated few HPT axis parameters, producing a small decrease in PVN-*Trh* mRNA levels [MS effect *F*_(1, 80)_ = 4.75, *p* = 0.03; Figure 4B] and MBH-*Trhde* mRNA levels [MS effect *F*_(1, 80)_ = 6.74, *p* = 0.01; Figure 4B] and lower serum T3 concentration than NH-chow males [MS effect *F*_(1, 80)_ = 13.3, *p* = 0.0005; Figure 4C]. The HFC diet induced changes depending on age of diet initiation and neonatal stress; NH-HFC30 male rats had lower serum T4 [diet effect *F*_(2, 80)_ = 4.83, *p* = 0.04; Figure 4C] and T3 concentration [diet effect *F*_(2, 80)_ = 4.27, *p* = 0.01; Figure 4C] than the NH-chow fed group. Compared to MS-chow-fed animals, MS-HFC30 males had higher PVN-*Trh* expression [MS and diet effect *F*_(2, 80)_ = 14.95, *p* < 0.0001; Figure 4B] and reduced serum concentration of TSH [diet effect *F*_(2, 80)_ = 6.05, *p* = 0.004; MS, diet and sex effect *F*_(2, 80)_ = 3.87, *p* = 0.026; Figure 4C], T4 [MS, diet and sex effect *F*_(2, 80)_ = 3.73, *p* = 0.02; Figure 4C], and T3 [diet effect *F*_(2, 80)_ = 4.27, *p* = 0.01; MS and diet effect *F*_(2, 80)_ = 8.62, *p* = 0.0004; MS, diet and sex effect *F*_(2, 80)_ = 3.87, *p* = 0.026; Figure 4C]. Different effects were observed in NH and MS male rats that had access to the HFC diet from Pd60; PVN-*Trh* expression and serum T4 concentration were lower in NH-HFC60 compared to NH-chow-fed animals (Figures 4B,C); in contrast, MS-HFC60 males had lower PVN-*Trh* and MBH-*Trhde* expression and serum TSH and T4 concentrations compared to MS-chow-fed rats (Figures 4B,C).

In female rats submitted to restraint, diet and neonatal stress effects were distinct from those observed in males. A decrease in mRNA levels of *Trhde* in MBH [sex effect *F*_(1, 80)_ = 3.73, *p* = 0.05; MS and sex effect *F*_(1, 80)_ = 10.57, *p* = 0.001; Figure 4E] and of serum TSH concentration [MS, diet and sex effect *F*_(2, 80)_ = 3.87, *p* = 0.026; Figure 4F] was observed in MS compared to NH rats. In NH rats, access to an HFC diet, either starting at puberty or adulthood, reduced MBH-*Trhde* [sex effect *F*_(1, 80)_ = 3.73, *p* = 0.05; MS and sex effect *F*_(1, 80)_ = 10.57, *p* = 0.001; diet and sex effect *F*_(2, 80)_ = 4.32, *p* = 0.04; Figure 4E] and *Dio2* mRNA levels [diet effect *F*_(2, 80)_ = 7.25, *p* = 0.001; sex effect *F*_(1, 80)_ = 4.3, *p* = 0.01; diet and sex effect *F*_(2, 80)_ = 6.36, *p* = 0.003; Figure 4E] compared to chow-fed females. In contrast, in MS rats, the HPT axis activity was altered in HFC30, but not in HFC60 rats compared to the chow-fed group: PVN-*Trh* expression was increased [sex effect *F*_(1, 80)_ = 14.22, *p* = 0.0004; MS, diet and sex effect *F*_(2, 80)_ = 5.46, *p* = 0.0065; Figure 4E], while MBH-*Dio2* expression was reduced [MS and sex effect *F*_(1, 80)_ = 6.17, *p* = 0.01; Figure 4E], and serum concentrations of T4 [MS, diet and sex effect *F*_(2, 80)_ = 3.73, *p* = 0.02; Figure 4F] and T3 increased [MS and sex effect *F*_(1, 8)0_ = 26.51, *p* < 0.0001; MS, diet, and sex effect *F*_(2, 80)_ = 3.24, *p* = 0.04; Figure 4F].

## Discussion

Nutrition and stress play a major role in programming neuroendocrine systems that regulate energy homeostasis and body weight (53), but whether early-life stress contributes to metabolic alterations later in life is controversial with evidence for both improved or worsened metabolic disease risk (3, 10). Our results support published data indicating that compared to NH rats, MS reduces food intake in both sexes at adulthood (8) and enhances serum corticosterone and insulin concentrations, but only in males (54–56), suggesting a degree of insulin insensitivity as in Mela et al. (57). An HFC feeding increased serum insulin concentration in MS males but reduced that of corticosterone, consistent with previous data (5). MS-induced insulinemia is prevented by mechanical and tactical stimulation of the pups (56), supporting the role of neonatal stress shown to alter the pancreatic beta-cell function and insulin sensitivity in rats, with males being more sensitive than females (58, 59).

During adolescence, development of feeding behavior is vulnerable to insults, especially in females (37). Some reports describe that constant availability of palatable foods increases female susceptibility to develop obesity and metabolic syndrome later in life (37, 38), exacerbated by MS in adult female rats (9). Our results did not reproduce these findings; MS did not potentiate obesity in adult HFC-fed females, in agreement with other studies (5, 8). Differences could be due to the type and composition of the diet (10). It is interesting to notice the consistency in the proportion of macronutrient intake of NH and MS male and female rats with diet choice, even after 3–4 months of exposure to HFC foods; although their carbohydrate and fat intake was high, rats maintained the minimum protein intake required for growth maintenance (51). The high proportion of fats and carbohydrates consumed by the NH-HFC and MS-HFC groups most likely reinforced energy intake through enhanced hedonic feeding (60, 61).

### MS and 1 Month of an HFC Diet Regulate the Basal Activity of the HPT Axis

Expression of MBH neuropeptides was evaluated due to their important role not only in food intake and energy balance but also in regulating the activity of PVN-TRH neurons (22). An HFC diet, but not MS, induced sex-specific changes in MBH-orexigenic and -anorexigenic peptides, with low *Npy* expression in NH-HFC males and elevated *Pomc* expression in NH-HFC females compared to chow-fed groups (49, 50). In contrast to previous reports of HPT axis activation with a high-fat saturated diet in male rodents (62–64), the HFC diet reduced serum TSH levels in NH males, coincident with increased expression of *Trhde*, supporting the lowering effect of a high-fat unsaturated diet on serum TSH concentration (65).

MS altered some parameters of the HPT axis activity only in males: expression of *Trhde* was higher in MS-HFC60 and MS-chow-fed rats than in NH animals, and serum TSH concentration decreased accordingly in MS-chow-fed rats as reported previously (32). NH females did show a slight activation of the HPT axis by an HFC diet, as serum T4 levels increased together with two activators of PVN-TRH neurons: serum leptin concentration and MBH-*Pomc* expression (22). Increased serum T4 could contribute to the enhanced expression of MBH-*Trhde* observed in NH-HFC females, as injection of this hormone increases the enzyme's expression in tanycytes (26). These changes, however, were not observed in MS females, in spite of having increased concentration of serum leptin.

### MS and 3–4 Months of an HFC Diet Regulate Metabolic and Stress Parameters in Acutely Restrained Rats

Obesity and chronic hyperglycemia are risk factors in developing metabolic derangements (i.e., reduced insulin sensitivity) (66–68). Adolescent male rats appear to be more sensitive to a high-fat saturated diet-induced obesity than adults, producing more drastic metabolic effects when a high-fat diet is administered to young males compared to adult-fed rats (69, 70). Contrary to these reports, consumption of an HFC diet from either adolescence or adulthood induced a similar obese state and moderate basal hyperglycemia in MS and NH male rats, suggesting reduced insulin sensitivity (71) whether the HFC diet was offered starting at adolescence or adulthood, although this remains to be confirmed. In addition, the HFC diet heightened the hyperglycemic response to restraint stress in males, and consequently, serum insulin levels were higher in comparison to chow-fed groups, although serum insulin concentration was slightly higher in NH-HFC60 males. In contrast, the HFC diet enhanced serum insulin concentration in restrained females (independently of MS), if initiated at adolescence but not at adulthood, despite lack of differences in basal blood glucose concentration and similar stress-induced hyperglycemia, suggesting insulin resistance in NH-HFC30 and MS-HFC30 females (71). These results support the idea that puberty is a vulnerable period for females with unrestricted access to palatable foods and could increase their risk to develop metabolic alterations in adulthood (37). Nevertheless, our results show that an HFC diet, but not neonatal stress, is a major determinant of serum metabolic markers after acute stress in a sex-specific manner.

The restraint-induced serum corticosterone response was higher in MS than in NH males (6, 11, 72), and the HFC diet dampened it, but only in NH rats as reported (16, 36, 73, 74). A sex dimorphism was evidenced in the effects of early-life stress since MS males' response to stress was independent of diet in contrast to MS females, which had lower *Crh* expression in HFC60 and corticosterone concentration in HFC30 and HFC60 than chow-fed rats. Furthermore, although NH females have higher basal corticosterone concentration, the extent of their response to acute stress was lower than that in NH males, in contrast to some reports (34, 35). Serum concentration of 17β-estradiol could have contributed to the sex differences observed in the HPA axis activity in acutely restrained rats and to the effect of palatable foods to reduce restraint stress responses (34, 75), since serum 17β-estradiol levels were reduced by an HFC diet in NH and MS females, as reported previously (76, 77). These results suggest that an HFC diet alters the HPA axis activity differently in male than in female rats, with MS being a contributing factor in males but not in females, and highlights the complex interaction of diet, gonadal steroids, and stress.

### MS and 3–4 Months of an HFC Diet Regulate HPT Axis Activity in Acutely Restrained Rats

Expression of arcuate peptides *Pomc* and *Npy* are regulated by the animal's energy state but also by stress (78–81). MBH-*Npy* expression was higher in MS-chow-fed restrained male and female rats than in NH-chow groups; neonatal stress *per se* does not affect *Npy* expression in male and female rats (5, 57, 82), but it increases hyperreactivity to restraint stress in adulthood (6, 11). As glucocorticoids (83) and restraint increase mRNA levels of *Npy* in the arcuate nucleus (albeit 24-h after initiated the stress) (78–80), the combination of MS and adult acute stress may have heightened *Npy* expression. Sex differences were observed in MBH neuropeptide expression in HFC-fed rats; *Npy* expression was higher in NH females that had access to the HFC from adolescence compared to NH-chow rats, but not in males, suggesting an altered diet-induced arcuate NPY activation and increased vulnerability of young females to chronic high-fat feeding (84). Restraint stress increases arcuate *Pomc* expression in adult male rats, although 4 h after stress (78, 79), its expression was inhibited in the MBH of NH-HFC30, MS-HFC30, and MSHFC-60 males compared to chow-fed rats, despite increased serum leptin levels, which is concordant with a hyperphagic behavior, a diet-induced obesity, and male rats' higher susceptibility to develop diet-induced leptin resistance compared to females (84–86). Thus, our results are in agreement with the reported impairment of the anorexigenic leptin–POMC system in males, whereas in females, there is an over-activation of the orexigenic NPY system in high-fat-fed rats (84).

Restraint and other forms of psychological stress rapidly inhibit HPT axis activity in adult male rats fed a standard diet and without a history of early-life stress (45, 46, 87). In contrast to the blunting effect of an HFC diet on restraint-induced *Crh* expression, compared to chow-fed rats, that of *Trh* was inhibited in NH-HFC60 and MS-HFC60 males as observed with *Pomc* expression. The restraint-induced decrease of serum TSH concentration in NH-chow males (46) was presumably blunted in MS-chow rats, most probably due to the lower expression of *Trhde* in MS in comparison to NH-chow males. These changes are not explained by altered serum TH or corticosterone concentration but could be due to lowered PVN-*Trh* expression since it has recently been reported that TRH may upregulate mRNA levels of *Trhde* and activity in tanycytes (88). Serum T4 concentration was lower in HFC- than in chow-fed males in both NH and MS groups, but did not coincide with changes in serum concentration of T3, which varied the most, as strong decreases were observed in MS-chow compared to NH-chow and in NH-HFC30 and MS-HFC30 groups compared to chow-fed animals.

Females responded differently to the acute stress situation dependent on diet initiation; NH-HFC30 and NH-HFC60 restrained females showed lower MBH expression of *Trhde* and *Dio2* without changes in PVN-*Trh*, serum TSH, or T3 concentration compared to NH-chow rats; it is tempting to speculate that acute stress modulated by an HFC diet regulates the expression of these enzymes, although this remains to be elucidated. Parallel changes of *Dio2* and *Trhde* expression are consistent with the proposal that T3 formed by D2 activates *Trhde* in MBH (26, 89). Even though *Dio2* expression and activity are not modified under basal conditions by a hypercaloric diet, as observed in our first experiment (Table 1) and other studies (62, 63), the combination of obesity with acute stress may have altered MBH-*Dio2* expression, since this enzyme is susceptible to various types of stimuli (47, 90–92). MS-HFC-fed females seemed to be less susceptible to stress than males, as reported previously (32). MS-HFC30 females had higher *Trh* expression and serum levels of T4 and T3 than MS-chow-fed rats, as well as decreased *Dio2* expression; this decrease may explain why expression of *Trh* was increased in MS-HFC30 animals in spite of a small increment in serum T4, not being sufficient to exert negative feedback on TRH at the PVN level (22, 93, 94). These changes were not observed in MS females that were exposed to an HFC diet until adulthood (HFC60), supporting females' susceptibility during adolescence, particularly to a diet that makes them more resilient to restraint stress.

## Conclusions

**Figure 5:**
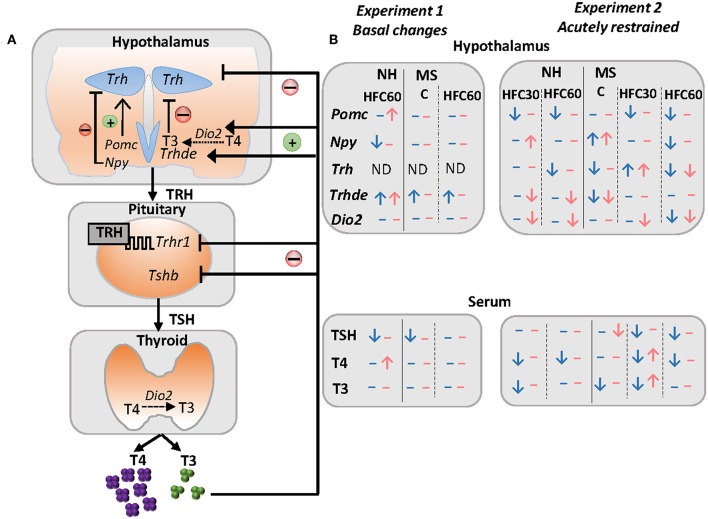
Schema of some peripheral inputs regulating the HPT axis and summary of changes of the HPT axis parameters and arcuate neuropeptides in response to maternal separation and 1–4 months of a high-fat/high-carbohydrate diet under basal conditions or after an acute stress in adult rats. **(A)** The HPT axis is controlled by thyrotropin-releasing hormone (TRH), synthesized in neurons of the hypothalamic paraventricular nucleus (PVN), and released at the median eminence near portal vessels that communicate with the anterior pituitary, where TRH activates its receptor (TRH-R1) increasing synthesis and release of thyrotropin (TSH) that controls the synthesis of thyroxine (T4) at the thyroid; T4 is converted to 3,3′,5-triiodo-L-thyronine (T3) by tissue deiodinases I or II (D1, D2). Released TRH at the median eminence may be degraded by the TRH-degrading ectoenzyme (TRH-DE) present in tanycytes before it travels to the pituitary, regulating the amount of TRH that reaches the thyrotrophs, and concomitantly, TSH and thyroid hormone synthesis and release, which in turn exert negative feedback at the pituitary and hypothalamus level. In addition, hypophysiotropic TRH neurons are negatively regulated by arcuate orexigenic neuropeptide Y (NPY), whereas anorexigenic neuropeptide pro-opiomelanocortin up-regulates *Trh* expression in the PVN. **(B)** Results summary of experiments 1 and 2. In experiment 1, we evaluated changes in maternal separated rats (MS) fed regular chow **(C)** vs. non-handled (NH), and the effect of 1 month of a high-fat/high-carbohydrate (HFC60) diet on NH and MS rats administered at Pd60. In experiment 2, we evaluated changes in NH and MS acutely restrained adult rats fed a C or an HFC diet starting at puberty (HFC30) or adulthood (HFC60) until postnatal day 160. NH and MS rats had free access to chow (C) or to an HFC diet from Pd30 or 60 until Pd160 and were restrained for 1 h before they were killed. Blue arrows and dashes represent males and pink denotes female rats; arrows indicate changes observed in NH-HFC30 and NH-HFC60 rats compared to the NH-C group; MS-C vs. NH-C; MS-HFC30 and MS-HFC60 compared to MS-C.

The HPT axis activity is regulated by energy-related cues (Figure 5A) as well as by stressors (Figure 5B). In this study, we show that MS did not exacerbate an obese phenotype or related metabolic alterations as hyperglycemia and insulinemia in either sex when rats have unrestricted access to an HFC diet composed mainly by unsaturated fatty acids. However, neonatal stress and/or long-term intake of an HFC diet altered the HPT axis activity in restraint-stressed rats in a sex-specific manner, and dependent on diet introduction at puberty or adulthood. Results are summarized in Figure 5B. Altogether, they emphasize the complex interaction of various environmental and physiological factors such as neonatal stress, nutrition, and sex in the regulation of the HPT axis activity, underlining the importance of studying sex differences, confirming that neuroendocrine responses to stress and nutrition are sexually dimorphic. Finally, our results are in line with clinical studies that report that different adult or adolescent psychopathologies due to childhood trauma are associated with alterations in thyroid function (29–31, 95, 96). The HPT axis is a dynamic and adaptive system; however, in this context, if adaptability is not met, psychological or metabolic pathologies may arise as observed in obese patients (97).

## Data Availability

All datasets generated for this study are included in the manuscript and/or the Supplementary Files.

## Ethics Statement

This study was carried out in accordance with the recommendations of the NIH guide for the care and use of laboratory animals and the Official Mexican Norm for production, care and use of laboratory animals NOM-062-ZOO-1999. The protocol and experiments were approved by the Bioethics Committee of Instituto de Biotecnología, UNAM, with authorization project number 311.

## Author Contributions

LJ-H performed the maternal separation and HFC diet protocol of both experiments, ELISA analyses, data analyses, and writing and text editing of the manuscript. FR performed the RNA extraction of hypothalamic tissues and RT-PCRs. J-LC contributed to manuscript editing. PJ-B contributed in the experimental design, data analyses, manuscript writing, and editing.

### Conflict of Interest Statement

The authors declare that the research was conducted in the absence of any commercial or financial relationships that could be construed as a potential conflict of interest.
