# Supplementary material for: Sex Dimorphic Responses of the Hypothalamus–Pituitary–Thyroid Axis to Maternal Separation and Palatable Diet
Source: Front Endocrinol (Lausanne). 2019 Jul 11;10:445. doi: 10.3389/fendo.2019.00445 (PMC6637657; doi:10.3389/fendo.2019.00445)
Supplement: Supplementary file 1 [file Data_Sheet_1.PDF]

MALES	*NH*			*MS*		
Pd 60-90	C	HFC30	HFC60	C	HFC30	HFC60
**Total Kcal/d**	65±2	95±3*	108±5*	59±1 $^{\eta}$	93±0.8*	80±3$^{*\&}$
**Kcal/d/Kg BW**	168±4.7	224±15.4*	251±13.6*	169±5.6	229±7.8*	213±4.7*
**FE (BWg/Kcal)**	5.9±0.3	5.45±0.4	5.38±0.4	5.87±0.5	5.39±0.26	5.53±0.2
**Carbohydrate (Kcal/d)**	37.4±0.9 (58%)	52±2* (55%)	54.5±3* (50.6%)	34.5±0.7 $^{\eta}$ (58%)	50±0.6* (53.7%)	37.8±1.7 (47.4%)
**Protein (Kcal/d)**	15.4±0.4 (24%)	12±0.3* (12.6%)	13.3±0.4* (12.4%)	14.3±0.3 $^{\eta}$ (24%)	12±0.1* (13%)	10.4±0.4* (13%)
**Fat (Kcal/d)**	11.6±0.3 (18%)	31±0.5* (32.4%)	39.9±1.4* (37%)	10.7±0.2 $^{\eta}$ (18%)	31±1.2* (33.3%)	31.4±1* (39.5%)
Pd 91-160						
**Total Kcal/d**	72±1	104±2*	113±3*	59±1 $^{\eta}$	93±3*	95±4*
**Carbohydrate (Kcal/d)**	41.5±0.7 (58%)	54±2* (52%)	54.1±1.6* (47.9%)	34.1±0.8 $^{\eta}$ (58%)	46±1.2* (49%)	42.8±1.4* (45%)
**Protein (Kcal/d)**	17.1±0.3 (24%)	13±0.2* (12.5%)	13.8±0.3* (12.2%)	14.1±0.3 $^{\eta}$ (24%)	12±0.2* (13%)	12±0.5* (12.7%)
**Fat (Kcal/d)**	12.8±0.2 (18%)	37±0.4* (35.5%)	45.2±1.7* (39.9%)	10.6±0.2 $^{\eta}$ (18%)	35±1* (38%)	40±1.6* (42.2%)
**Lys**	0.2±0.004	0.08±0.001*	0.09±0.005*	0.17±0.004 $^{\eta}$	0.07±0.003*	0.08±0.004*
**Thr**	0.14±0.002	0.06±0.0007*	0.08±0.003*	0.11±0.002 $^{\eta}$	0.05±0.002*	0.06±0.003*
**Trp**	0.04±0.008	0.02±0.0002*	0.02±0.0009*	0.03±0.0009 $^{\eta}$	0.02±0.0006*	0.02±0.001*
**Leu**	0.24±0.004	0.12±0.001*	0.16±0.005*	0.2±0.005 $^{\eta}$	0.11±0.003*	0.13±0.006*
**Met+Cys**	0.22±0.004	0.1±0.0008*	0.08±0.004*	0.18±0.003 $^{\eta}$	0.09±0.003*	0.11±0.005*
FEMALES	*NH*			*MS*		
Pd 60-90	C	HFC30	HFC60	C	HFC30	HFC60
**Total Kcal/d**	58±1^{A}	98.4±8*	73±1^{*A}	53±2 ^{A}	100.4±6*	79±8*
**Kcal/d/Kg BW**	267±9.3 ^{A}	334±32.7^{*A}	294±10.2^{*A}	259±6 ^{A}	392±30^{*A}	311±29.8^{*A}
**FE (BWg/Kcal)**	2.53±0.34 ^{A}	3.12±0.8 ^{A}	3.05±0.35 ^{A}	2.39±0.18 ^{A}	2.110.3 ^{A}	3.13±0.34 ^{A}
**Carbohydrate (Kcal/d)**	33.3±0.64 (58%)	52.3±4.2* (53%)	34.5±1.5* (47.2%)	31±0.9 $^{\eta}$ (58%)	50.2±3.8* (50%)	38±4.6 * (46.5%)
**Protein (Kcal/d)**	13.8±0.26 (24%)	13.4±1* (14%)	9.2±0.1* (12.6%)	12.8±0.36 $^{\eta}$ (24%)	13.9±0.8* (14%)	9.8±1.08 * (12.7%)
**Fat (Kcal/d)**	10.3±0.19 (18%)	32.5±3* (33%)	29.4±0.7* (40.2%)	9.6±0.27 $^{\eta}$ (18%)	36.3±2* (36%)	30.8±2.7 * (40.8%)
Pd 91-160						
**Total Kcal/d**	53±1 ^{A}	81.8±9* ^{A}	73±3* ^{A}	47±0.8 ^{A}	75±3* ^{A}	78±8* ^{A}
**Carbohydrate (Kcal/d)**	30.6±0.744 (58%)	44.5±4.2* (54.4%)	36±2.1* (49.5%)	27.3±0.46 $^{\eta}$ (58%)	35.8±2.2* (47.7%)	36.7±3.8* (47.3%)
**Protein (Kcal/d)**	12.6±0.3 (24%)	10.4±0.9* (12.7%)	8.9±0.4* (12.3%)	11.3±0.2 $^{\eta}$ (24%)	10.3±0.3* (13.7%)	9.8±0.9 (12.7%)
**Fat (Kcal/d)**	9.5±0.23 (18%)	26.9±3.3* (32.8)	27.7±0.99* (38.1%)	8.4±0.14 $^{\eta}$ (18%)	28.9±0.4* (38.5%)	30.9±3* (39.9%)
**Lys**	0.15±0.003	0.06±0.004*	0.06±0.003*	0.13±0.002 $^{\eta}$	0.07±0.001*	0.07±0.007*
**Thr**	0.1±0.002	0.05±0.004*	0.05±0.002*	0.09±0.001 $^{\eta}$	0.05±0.001*	0.05±0.006*
**Trp**	0.034±0.0008	0.01±0.001*	0.015±0.0007*	0.03±0.0005 $^{\eta}$	0.01±0.0004*	0.01±0.001*
**Leu**	0.18±0.004	0.09±0.008*	0.09±0.004*	0.16±0.003 $^{\eta}$	0.1±0.001*	0.1±0.01*
**Met+Cys**	0.16±0.003	0.06±0.003*	0.05±0.003*	0.14±0.0009 $^{\eta}$	0.06±0.002*	0.06±0.005*

Supplementary table 1. Effects of maternal separation and three-four months of a palatable diet on Kcal intake, macronutrients and some essential amino acids. Non-handled (NH) or maternal separated (MS) male and female rats were offered either chow (C) or a high-fat/high-carbohydrate diet (HFC) at postnatal day 30 (Pd30) or 60 (Pd60) *ad libitum* until Pd160. Macronutrients are calculated as the average of Kcal intake per day (Kcal/d) and values in parenthesis represent the % of total Kcal/d consumed of the respective macronutrient. Intake of essential amino acids were calculated considering digestibility and bioavailability of protein and are presented as g/kg of diet. Data are presented as mean ± S.E.M. and were analyzed by a three-way ANOVA and to assess differences between groups the Holm-Sidak multiple comparisons test was used, $p < 0.05$: $^{\eta}$ *vs* NH-chow, * *vs* respective chow-fed group, $^{\&}$ *vs* HFC30 same treatment group, ^{A} sex effect.
