# Supplementary figures and images for: Sex Dimorphic Responses of the Hypothalamus–Pituitary–Thyroid Axis to Maternal Separation and Palatable Diet
Source: Front Endocrinol (Lausanne). 2019 Jul 11;10:445. doi: 10.3389/fendo.2019.00445 (PMC6637657; doi:10.3389/fendo.2019.00445)

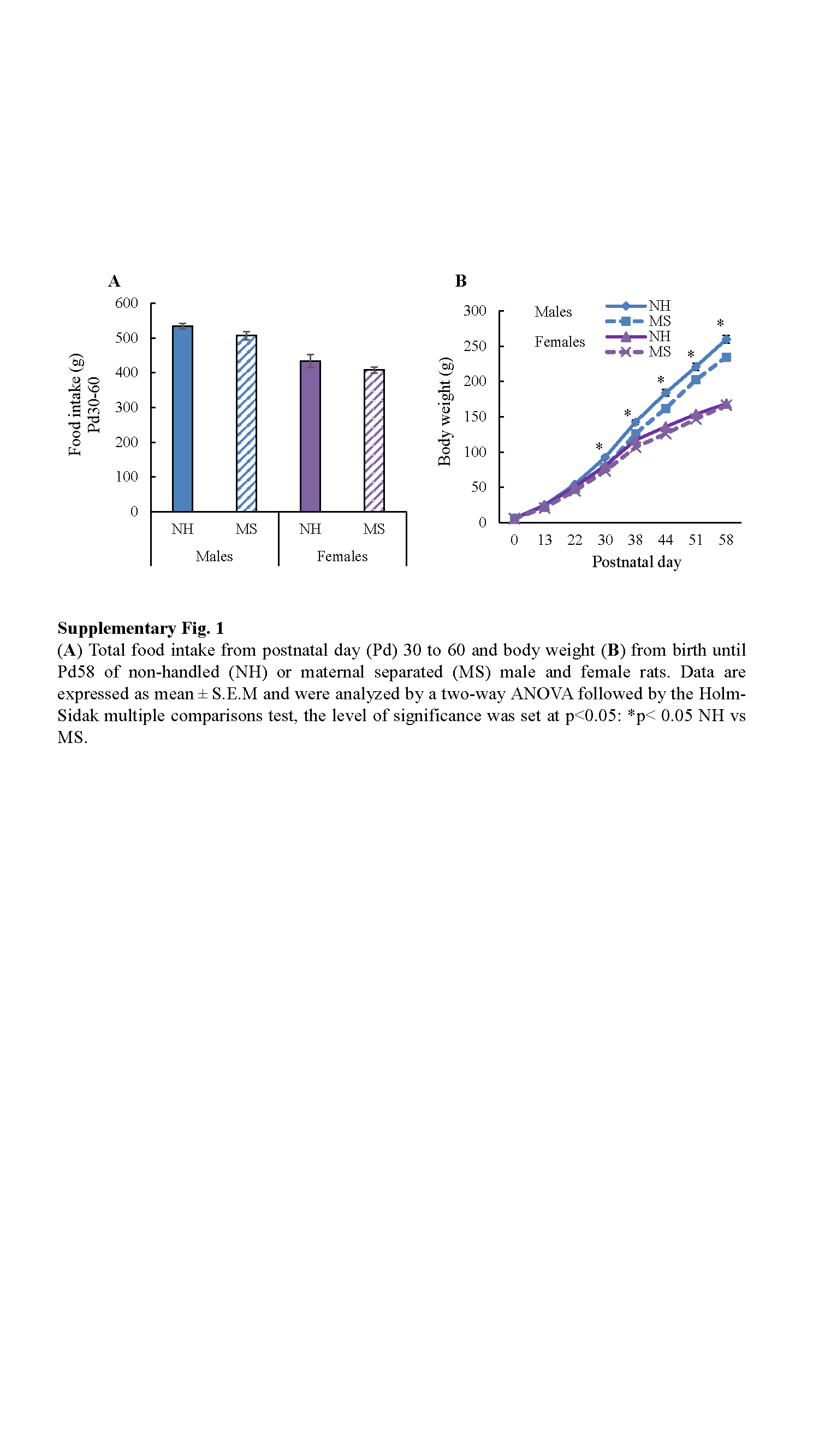

Supplement: Supplementary file 2 [file Image_1.TIFF]

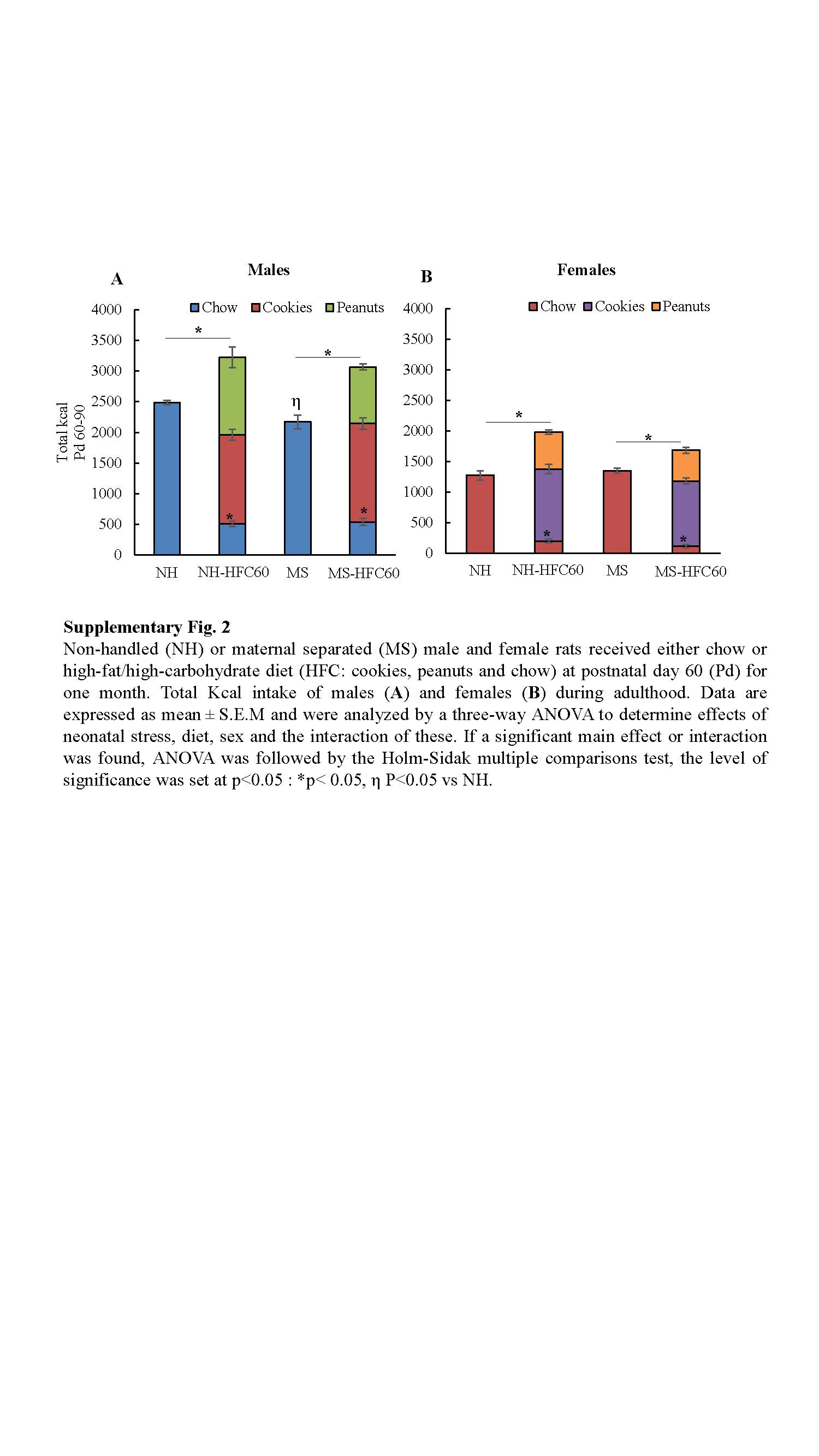

Supplement: Supplementary file 3 [file Image_2.TIFF]

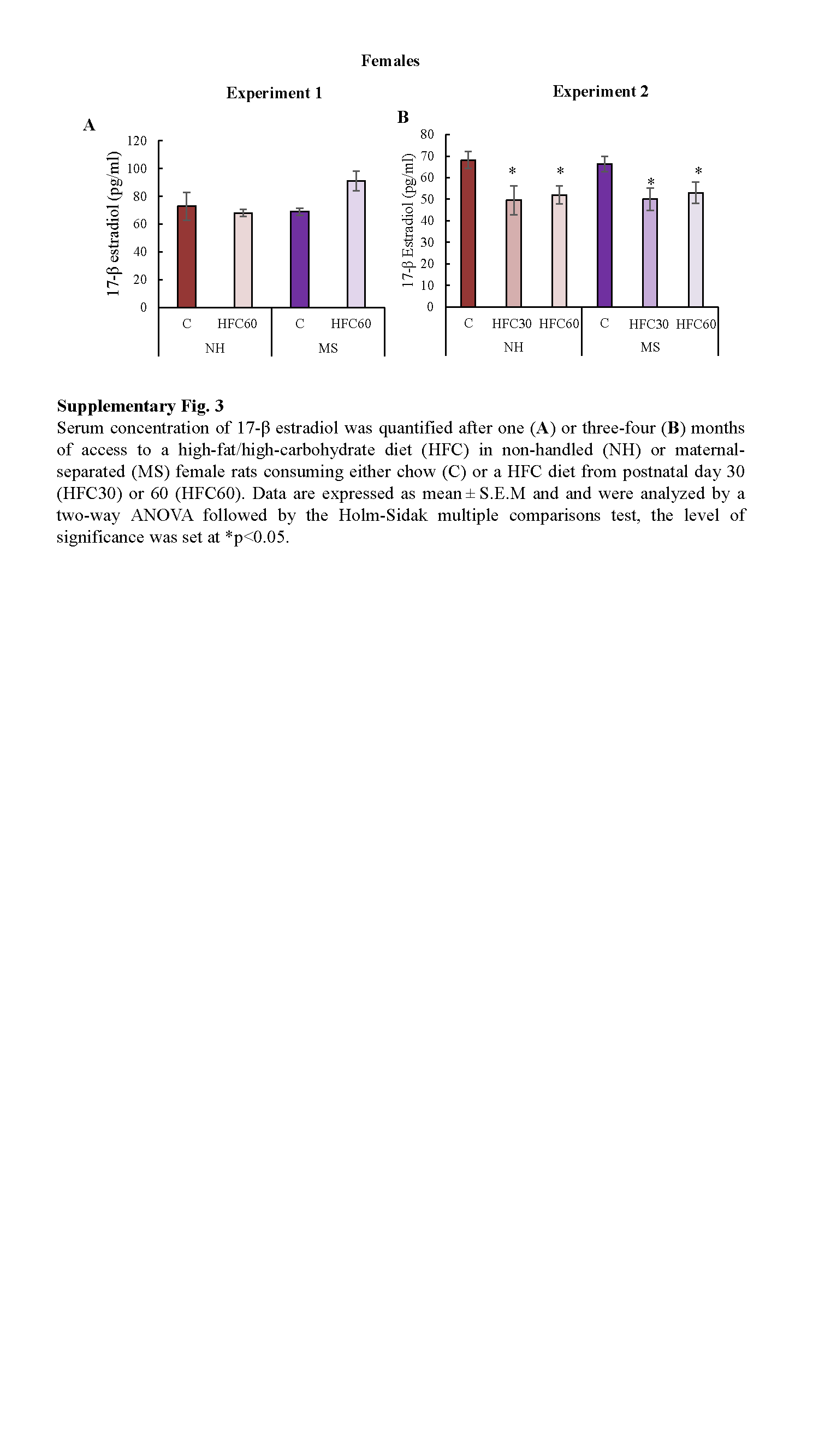

Supplement: Supplementary file 4 [file Image_3.TIFF]

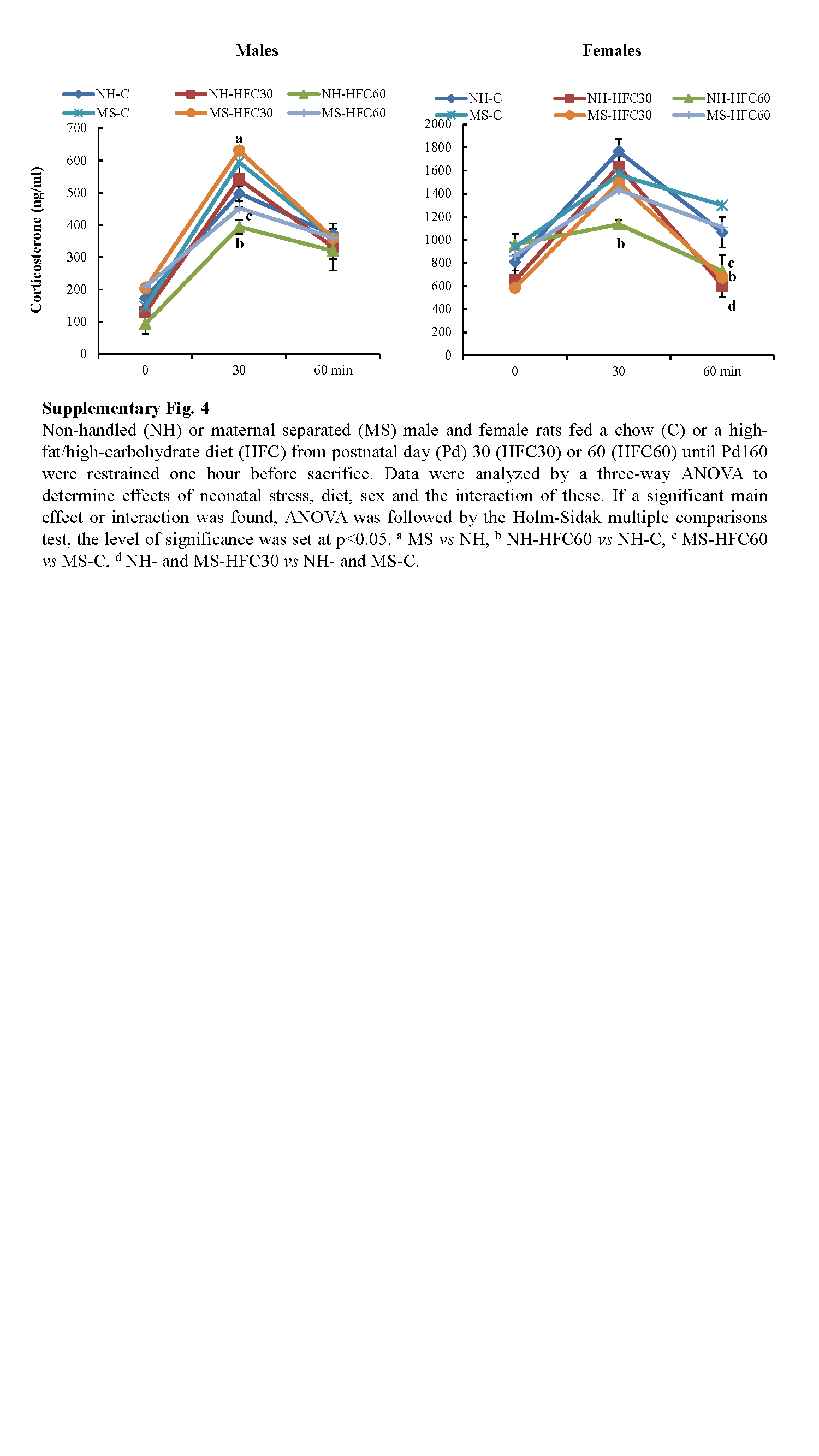

Supplement: Supplementary file 5 [file Image_4.TIFF]
